# Draft Genome Sequence of a *Chitinophaga* Strain Isolated from a Lignocellulose Biomass-Degrading Consortium

**DOI:** 10.1128/genomeA.01056-16

**Published:** 2017-01-19

**Authors:** Luciano T. Kishi, Erica M. Lopes, Camila C. Fernandes, Gabriela C. Fernandes, Lais P. Sacco, Lucia M. Carareto Alves, Eliana G. M. Lemos

**Affiliations:** a Departamento de Tecnologia, Universidade Estadual Paulista (UNESP), Faculdade de Ciências Agrárias e Veterinárias, Laboratório de Bioquímica de Microrganismos e Planta (LBMP), Jaboticabal, São Paulo, Brazil; b Departamento de Tecnologia, Universidade Estadual Paulista (UNESP), Faculdade de Ciências Agrárias e Veterinárias, Laboratório Multiusuário de Sequenciamento em Larga Escala e Expressão Gênica, Jaboticabal, São Paulo, Brazil

## Abstract

*Chitinophaga* comprises microorganisms capable of degrading plant-derived carbohydrates, serving as a source of new tools for the characterization and degradation of plant biomass. Here, we report the draft genome assembly of a *Chitinophaga* strain with 8.2 Mbp and 7,173 open reading frames (ORFs), isolated from a bacterial consortium that is able to degrade lignocellulose.

## GENOME ANNOUNCEMENT

The *Chitinophagaceae* family was first described in 1981 by Sangkhobol and Skerman (1, 2) as being Gram-negative bacteria that were filamentous and with motility, originating from pine forest leaf litter (3). At the time of writing, the genus *Chitinophaga* comprises 12 recognized species (4). Brazilian soils with sugarcane culture have many species of the *Chitinophagaceae* family (4, 5), and an isolate from a bacterial consortium degrading lignocellulose isolated from sugar cane soil was used as study material.

Genomic DNA from isolate CB10 was extracted using the Wizard genomic DNA purification kit (Promega). *De novo* sequencing of CB10 was carried out using the Ion Torrent (Thermo Fisher Scientific, USA). The library was constructed using the Ion Xpress Plus fragment library kit (Thermo Fisher Scientific), under conditions recommended by the manufacturer. Single-read sequencing (1 × 200 bp) was performed on an Ion Torrent proton sequencer (Thermo Fisher Scientific) on a separate PI chip, according to the manufacturer’s protocols.

The analysis of the result of CB10 sequencing was able to identify a partial genome of a *Chitinophagaceae* family bacterium with a total of 59,136,612 reads sequenced, which had 22,401,727 reads with a Phred quality greater than 20 identified using Prinseq (http://prinseq.sourceforge.net). The organization of contigs was performed using the SPAdes program (6), resulting in 6,041 contigs (8,209,897 bp), 50% G+C content, 7,173 annotated open reading frames (ORFs), and 127 RNAs. Gene prediction and functional annotation of the draft genome were performed using the RAST server (7).

The annotated genome shows ORFs for various systems: cell wall and capsule (158 ORFs), virulence, disease, and defense (145 ORFs), potassium metabolism (19 ORFs), phages, prophages, transposable elements, and plasmids (8 ORFs), membrane transport (186 ORFs), iron acquisition and metabolism (16 ORFs), RNA metabolism (169 ORFs), protein metabolism (243 ORFs), cell division and cell cycle (41 ORFs), regulation and cell signaling (47 ORFs), secondary metabolism (24 ORFs), DNA metabolism (116 ORFs), dormancy and sporulation (six ORFs), stress response (119 ORFs), sulfur metabolism (48 ORFs), phosphorus metabolism (38 ORFs), and central carbohydrate metabolism (170 ORFs).

The partial genome analysis shows 75% similarity to the genome of a *Chitinophaga* strain from the NCBI databases (4).

### Accession number(s).

The draft genome sequence has been deposited in NCBI GenBank under the accession number MLAV00000000.
